# Exotic species invasions undermine regional functional diversity of freshwater fish

**DOI:** 10.1038/s41598-019-54210-1

**Published:** 2019-11-29

**Authors:** Marco Milardi, Anna Gavioli, Janne Soininen, Giuseppe Castaldelli

**Affiliations:** 1 University of Ferrara, Department of Life Sciences and Biotechnology, via Luigi Borsari 46, 44121 Ferrara, Italy; 2 University of Helsinki, Department of Geosciences and Geography, PO Box 64, 34 – 38 Bowen Street, FI-00014 Helsinki, Finland; 3 Present Address: Fisheries New Zealand - Tini a Tangaroa, Ministry for Primary Industries - Manatū Ahu Matua, 34 – 38 Bowen Street, Wellington, New Zealand

**Keywords:** Biodiversity, Community ecology, Freshwater ecology, Invasive species

## Abstract

Exotic species invasions often result in native biodiversity loss, i.e. a lower taxonomic diversity, but current knowledge on invasions effects underlined a potential increase of functional diversity. We thus explored the connections between functional diversity and exotic species invasions, while accounting for their environmental drivers, using a fine-resolution large dataset of Mediterranean stream fish communities. While functional diversity of native and exotic species responded similarly to most environmental constraints, we found significant differences in the effects of altitude and in the different ranking of constraints. These differences suggest that invasion dynamics could play a role in overriding some major environmental drivers. Our results also showed that a lower diversity of ecological traits in communities (about half of less disturbed communities) corresponded to a high invasion degree, and that the exotic component of communities had typically less diverse ecological traits than the native one, even when accounting for stream order and species richness. Overall, our results suggest that possible outcomes of severe exotic species invasions could include a reduced functional diversity of invaded communities, but analyzing data with finer ecological, temporal and spatial resolutions would be needed to pinpoint the causal relationship between invasions and functional diversity.

## Introduction

Biodiversity is a key element of ecosystem functioning and characterizes its resilience to different pressures^1^, but suffers from a general worldwide decline^2^. Among the causes of global biodiversity loss, exotic invasions are often placed at the top^3,4^, but mechanisms and impact of species invasions might vary in different ecosystems, taxa and spatial scales. While a lot of attention has been devoted to the consequences of exotic invasions on species taxonomic diversity at different geographical scales^5^, hardly any regional extinctions have been recorded in aquatic taxa such as fish (with some notable exceptions, e.g.^6,7^), but extirpation, species substitution and decrease of native biomass have all been reported as a local result of exotic invasions^8–11^. The detection of exotic invasions‘ effects could thus depend on the spatial and biological detail level of the data used, so that some effects could be potentially overlooked or misinterpreted if the data is insufficiently detailed.

For example, fish introductions could enrich functional diversity at the regional scale, because, from a presence/absence perspective, they can increase regional species richness and consequently increase the number of functional traits present in that area^12^. Functional diversity describes the distinctive assemblage of morphological, biochemical, physiological, structural, phenological or behavioral traits that characterizes living communities, and that is highly coupled to environmental conditions^13^. Ecofunctional diversity is a subset of functional diversity, focusing on the combination of ecological traits in communities^14^, but is referred to simply as functional diversity hereafter, because it is a more common term. Ecological traits in a community are usually selected by factors such as habitat diversity, geography, land use or water chemistry^15^, but established exotic species are often generalists^16^ and invasion dynamics (e.g. human-aided dispersion) could partly override habitat selectivity.

Competition with introduced exotic species could act as an additional filter for native species, sometimes stronger than environmental gradients^11^. Furthermore, widespread and severe biological invasions could result in the taxonomic homogenization of invaded communities^17–19^, with few exotic species dominating heavily invaded areas, a pattern which has been detected at least in plants^20^. Most likely, taxonomic homogenization would also affect functional diversity of invaded areas, as it leads to communities with a lower number of species and a lower variety of traits, but it remains unclear whether this is a significant element of exotic invasions.

Exotic species are a main cause of the loss of biological diversity in the Mediterranean region^21,22^, particularly in freshwater habitats^23^. The functional structure of Mediterranean freshwater fish communities is relatively uncharted, and an ecological trait characterization of native and exotic fish species has only been recently defined for some areas^24–26^. This finally enables further research on the linkages between biological invasions, functional diversity and the environment.

We used a spatially-broad yet very detailed dataset, comprising several river basins in northern Italy at a late invasion stage (i.e. >30 years after major invasions), as a test case to explore the outcomes of exotic species invasion in freshwater fish communities and to investigate the relationships between environmental factors, invasions and functional diversity. We used boosted regression tree (BRT) analysis to test the hypothesis (H_1_) that functional diversity of exotic and native species would respond differently to environmental variables, as invasion dynamics could temporarily override habitat selectivity. We then used spatial and regression analyses to test whether (H_2_) the overall functional diversity of communities would be negatively or positively affected by different degrees of exotic invasions. Our results would ultimately reveal the connections between functional diversity and exotic species invasions, on the background of habitat filtering.

## Results

**Figure 1:**
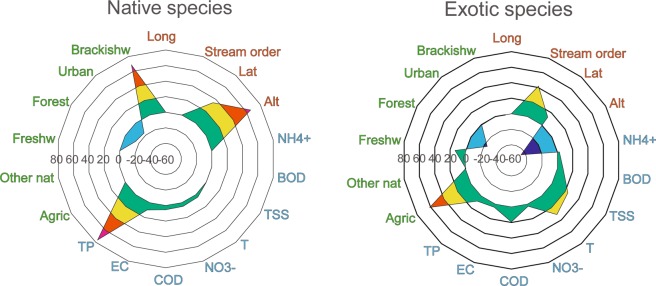
Boosted Regression Tree (BRT) summary showing the relative influence of geographical variables (in orange), water physico-chemical variables (in blue) and land use (in green) on freshwater fish functional diversity (calculated through the functional dispersion, FDis metric, applied to the ecological traits of species) for native (left panel) and exotic (right panel) species. The variable abbreviations stand for: *Long* – longitude, *Lat* – latitude, *Alt* – altitude, *NH*_4_^+^ – ammonia, *BOD* – biological oxygen demand, *TSS* – total suspended solids, *T* – water temperature, *NO*_3_ – nitrate nitrogen, *COD* – chemical oxygen demand, *EC* – electrical conductivity, *TP* – total phosphorus, *Agric* – agricultural, *Other nat* – other natural area, *Freshw* – freshwater, *Forest* – forest, *Urban* – urban and *Brackishw* – brackish water.

BRT analysis showed that environmental variables typically had different magnitude and direction of relative influence on exotic and native species functional diversity (Fig. 1). While altitude was one of the most significant variables negatively affecting native species functional diversity (and positively that of exotic ones), high temperature, low salinity or high turbidity were clearly linked to higher exotic species functional diversity (Fig. 1). Forest cover was associated to lower functional diversity of both native and exotic species, while brackish water was associated to a higher functional diversity of native species (Fig. 1).

**Figure 2:**
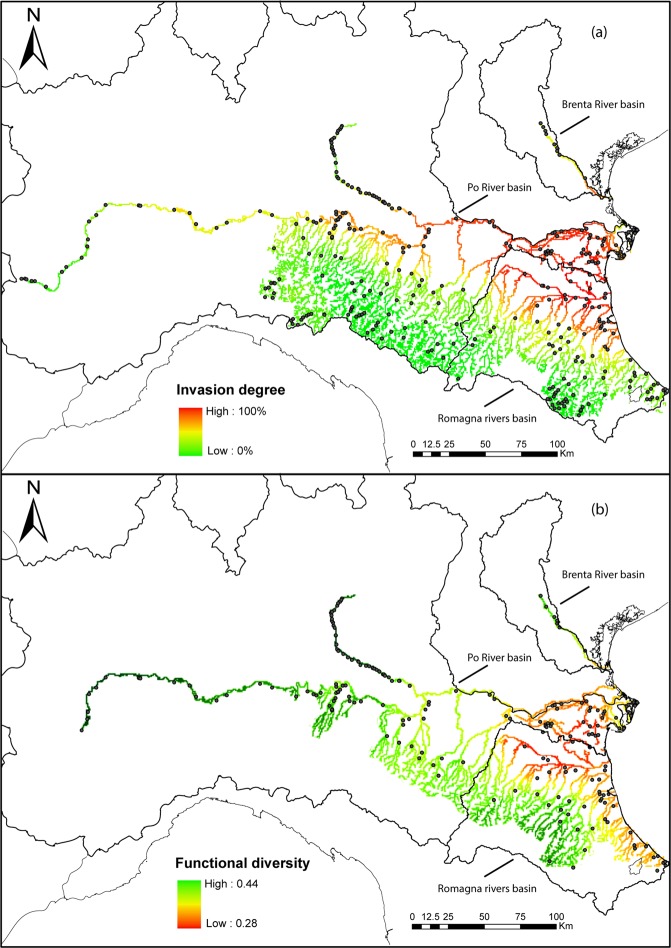
(**a**) Invasion degree in freshwater fish communities of northern Italy, and (**b**) spatial distribution of functional diversity (calculated through the FDis metric applied to the ecological traits of species) in the invaded sites, obtained by linear kriging. Dots represent the sampling sites used in each analysis. Colors represent the severity of invasion (% of exotic species abundance in a community) and the functional diversity of communities, respectively.

Several sites showed minimum levels of exotic invasion (invasion degree ≤ 10% for 140 sites, 41.9% of the total) and most of these sites hosted completely native communities (126 sites, 37.6% of the total). However, the majority of sites were invaded (209 sites, 62.4% of the total) and the invasion degree was relatively severe in most of them (invasion degree ≥ 50% for 134 sites, 40% of the total), including some sites where the community exclusively comprised exotic species (10 sites, 3% of the total). Our spatial analysis underlined that the most severely invaded sites were mostly located on the lower stretches of most watercourses examined (Fig. 2a). Moreover, in invaded communities, there was a clear spatial overlap between the most invaded areas and the areas where functional diversity was lowest (Fig. 2b).

**Figure 3:**
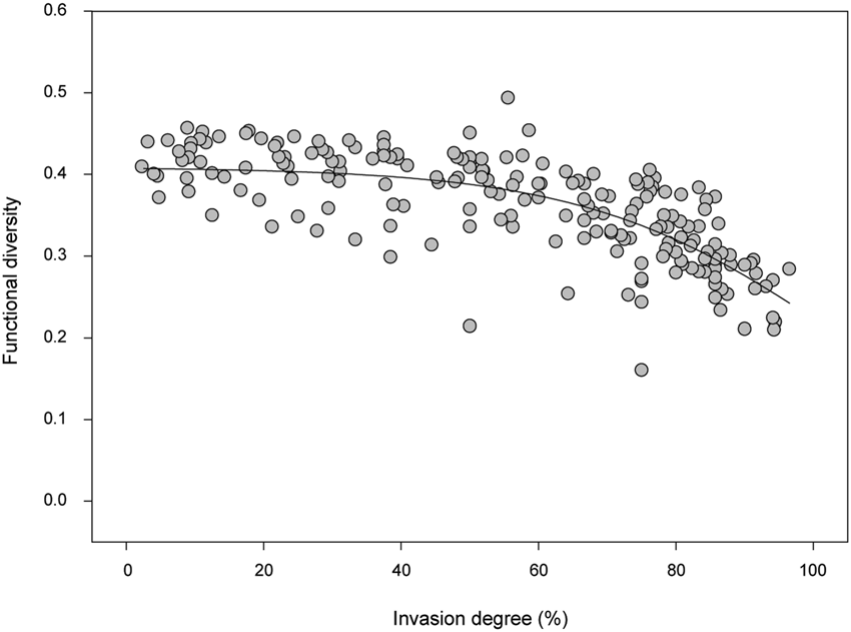
Italian freshwater fish communities’ functional diversity (calculated through the FDis metric applied to the ecological traits of species), along the invasion gradient. Circles represent values of overall functional diversity for each invaded site, while the line represents the best-fitting non-linear regression line (see Supplementary Table 3 for best fit regression evaluation).

In invaded sites, functional diversity was also clearly negatively linked to the invasion degree (Fig. 3). At the highest invasion degrees, a decrease of nearly 50% of functional diversity, compared with less disturbed communities, could be observed.

**Figure 4:**
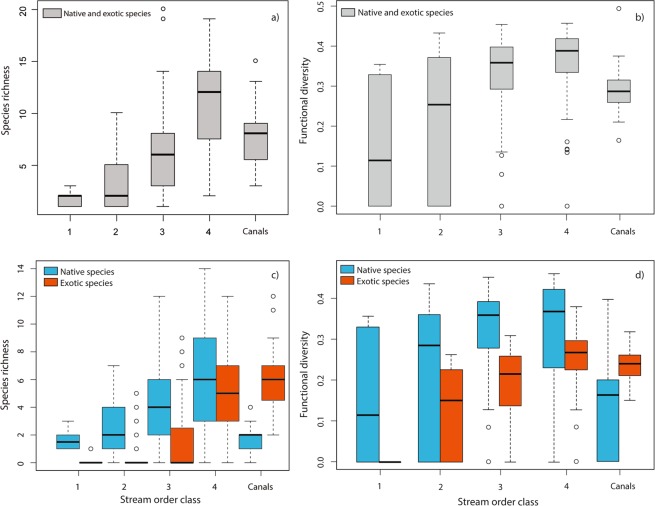
(**a**) Distribution of species richness and (**b**) functional diversity (calculated through the FDis metric applied to the ecological traits of species) by stream order classes, calculated on the whole fish community. The same patterns for species richness (**c**) and functional diversity (**d**) are also represented for exotic and native fish species separately. The horizontal bars in the boxes represent the median, the boxes’ hinges represent the first and third quartile, and the notches represent the 95% confidence interval of the median.

Low-order streams (in the uplands) showed lower values of species richness and functional diversity than high-order streams (in the lowlands) (Fig. 4a,b). Species richness was low in upland streams, but these habitats showed a larger variation in functional diversity than lowland streams. Canals, in the lowlands, had lower richness and functional diversity values than natural rivers in the same area (Fig. 4a,b). The difference between native and exotic functional diversity was consistently lower than what suggested by their relative richness (Fig. 4c,d), with native species usually showing higher values of functional diversity relative to their richness. Exotic species showed lower functional diversity values than native species in all natural rivers, which was most evident in higher order streams, but the pattern was reversed in canals (Fig. 4d).

**Figure 5:**
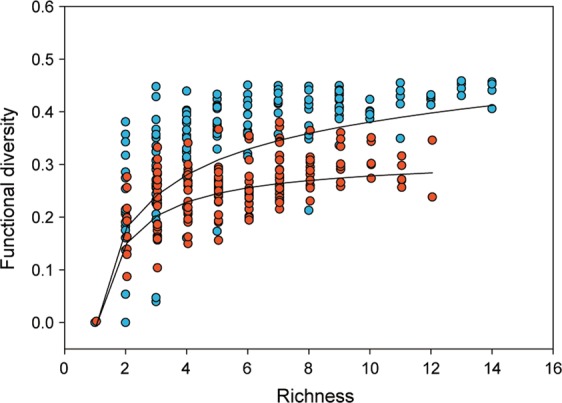
Native (blue circles) and exotic (red circles) functional diversity (calculated through the FDis metric applied to the ecological traits of species), in fish communities along a species richness gradient. Black lines represent best fitting non-linear regression lines for each distribution (see Supplementary Table 3 for best fit regression evaluation).

Both native and exotic functional diversity in communities were linked to species richness and tended to saturate with growing richness (Fig. 5), albeit with different rates and asymptotes. However, accounting for species richness, community functional diversity for exotic species was generally lower than that of native ones (Fig. 5).

## Discussion

Functional diversity of native and exotic species, as expressed by the FDis metric, responded similarly to most environmental constraints. However, confirming our first hypothesis (H_1_), we found significant differences in the effects of altitude (positive effect for native and negative effect for exotic species) and in the different ranking of constraints. This suggests that invasion dynamics could play a role in overriding some major environmental drivers of functional diversity. Our results also showed that a high invasion degree corresponded to a lower functional diversity of fish communities, confirming our second hypothesis (H_2_). Exotic species typically had a lower functional diversity than native species, even when accounting for stream order and species richness, which could be at the root of the observed lower functional diversity of highly invaded communities. Our results thus suggest that possible outcomes of severe exotic species invasions could include a reduced functional diversity of freshwater fish communities, and that analyzing data with finer ecological and spatial resolutions could help further investigate these effects.

The different ranking of environmental drivers for exotic and native species might indicate that there are some differences in the relevance of factors shaping their respective functional diversity patterns. For example, our BRT analysis highlighted the role of native marine species entering brackish habitats in contributing to the fish functional diversity in these areas. Our analysis results also suggested that geographical factors like altitude could play a major role to control invasion dynamics, as altitude appeared to be the main factor of divergence for native and exotic functional diversity. In addition to temperature, altitude is linked to a number of other factors, including habitat fragmentation but also habitat quality. Mountain streams typically have lower habitat complexity and resources availability but higher habitat quality than rivers in the lowlands^27,28^. However, upland streams can be more fragmented due to the presence of dispersal barriers^29,30^.

The high degree of invasion in the lower stretches of all basins also implies that lowland areas constitute a massive reservoir of exotic species, which could exert a significant propagule pressure towards higher altitudes^31,32^. This pressure is held in check, at least in some cases, by impassable barriers to migration and by unfavorable ecological conditions in upstream areas^33^. Habitat fragmentation and other consequences of water abstraction for human use are a worldwide issue often deeply interlinked with exotic species invasions^34,35^, freshwater communities functional diversity^36^, and homogenization processes^37^. Unfortunately, habitat connectivity restoration near heavily invaded areas may not be sufficient to restore native biodiversity, as it could favor exotic invasions in upstream areas. Furthermore, some fish species can colonize upstream areas through human-mediated transport even if migration barriers are not removed^38,39^. Our analysis did not show sharp regional invasion gradients in several non-fragmented rivers, albeit the spatial resolution of our data should have been sufficient to underline habitat fragmentation at that scale. Overall, exotic species invasion was still halted in upland areas, and we can only speculate that this could be attributed to natural gradients (e.g. temperature and habitat factors).

Perhaps the most immediate outcome of our work is a visualization of the degree of exotic freshwater fish invasion on a broad geographical scale, which highlighted the alarming decline in native species richness and abundance at most sites in the lowlands of our focus area. A high degree of invasion corresponded also to a lower functional diversity of invaded communities. Canals were among the most heavily invaded areas and a good example of this mechanism, with lower overall functional diversity than rivers in the same geographical area. This could be the result of simplified habitats, but also of the native species extinctions in this area, where only few natives remained at the time of sampling^9–11^, but still showing higher functional diversity for the few remaining native species than for the more abundant exotic ones. Lowland rivers with high-stream order class had generally more heterogeneous habitats than canals, offering more spatial and trophic niche options to species and thus presumably allowing for a higher overall functional diversity, yet showed a similar functional diversity to that detected in canals. This pattern could potentially be caused by the presence of exotic species, which have led to local homogenizations of the fish fauna in lowland rivers and canals that are connected to them. Conversely, stream order 2 sites had a very low invasion degree (at the extreme left of Fig. 3), yet the functional diversity of these communities was not much higher than more invaded sites. This perhaps indicates that invasion in these areas is not at the initial stages, and has caused a very moderate decline (if any) of native species, thus resulting in communities where the moderate loss is compensated by a corresponding increase in functional diversity provided by exotic species. Invasion in these areas probably did not progress further because of environmental constraints (e.g. water temperature or habitat) or competition with native species^11^.

Although there are only few documented cases of extinctions in freshwater fish^6–8^, previous studies in this area have highlighted how freshwater fish invasions could substitute native with exotic species, resulting in a decrease of native taxonomic diversity at the local level, especially when abundances are taken into account^9,10^. Taxonomic and functional substitutions have been previously advocated as non-detrimental or even beneficial for the environment and biodiversity^40,41^. However, from a functional perspective, our data show that exotic species generally had a lower functional diversity than native species across similar habitats, which was evident in higher order streams and in canals. As a consequence, substitution of native species with exotic ones could decrease the overall functional diversity of the community.

It has been previously suggested that the availability of habitat and trophic niches in the environment might be the ultimate factor that defines a limit to the possible expression of functional diversity. In this framework, if more species are added to the system functional diversity should not be positively affected, but rather show an asymptotic saturation of the available functional space^42^. While our results clearly show this asymptotic saturation, they also show that, in invaded communities, the functional diversity of exotic species was generally lower than native ones, perhaps because fewer exotic species with a limited number of traits are introduced. This could be at the root of the decrease observed in overall functional diversity of the highly invaded communities (>50% invasion degree), as the exotic component becomes predominant and drives the overall functional diversity of the community. This also suggests that a relatively smaller number of species with similar traits could be leading the invasion in the lowlands, and that high degrees of invasion might coincide with higher faunal homogenization i.e. Figure 3 of the present work,^11,19,43^. Our view is also supported by the relatively higher functional diversity of native species in the lowlands, despite their richness not being higher than exotic ones^44^. A possible explanation is the longer evolution history in the area, which allowed natives to be more functionally diverse and match the ecological niches in their native environment more closely^45^, albeit no studies have dealt with this aspect in fish communities, so far. Thus, our results clearly indicate that the overall functional diversity of highly invaded communities might be diminished, resulting in an overall heavier loss of diversity and adding a new dimension to the effects of exotic invasions, which should be of great interest to conservation science and management but needs to be further investigated. Our results disagree with the outcomes of the study by^12^, conducted at the macro-regional scale. This difference could stem from our different approach to the same problem: we analyzed a set of sites at a late-invasion stage rather than using a pre/post invasion approach, but also used species abundance measures and a finer spatial scale, which were advocated by^12^. The divergence in our outcomes indicates that different spatial and biological resolutions, and different study setups, deeply affect the results of the analysis (see e.g.^46^). It is also possible that the number and quality of functional traits investigated could further modulate the ultimate outcome of the analysis, suggesting that the role of these factors in affecting the results would need to be tested by future studies.

Given our results, we encourage further studies that deal with functional diversity and, more in general, a wider use of functional approaches before a consensus is formed on the ultimate effects of exotic invasions on diversity. Spatially- and temporally-detailed measures of species abundances would be crucial in these studies, as subtler changes in abundances could be detected before regional extinctions occur. Future studies should investigate in further detail whether a true functional loss has occurred, using a wider geographical scale and comparing areas with different invasion degrees (or theoretical reference communities). Furthermore, large-scale patterns of community diversity could be explored using measures of relative functional diversity, to express the amount of functional diversity expressed by an average species unit. We also encourage studies that explore these mechanisms in different taxa and, whenever possible, compare results across taxa. Ultimately, other aspects of functional diversity should also be investigated, as they might provide useful insights on the final outcomes of exotic and native species interactions. These are all necessary elements to fully understand the ecological consequences of functional diversity loss and its significance for ecosystem functioning at large.

## Materials and Methods

### Study area

Northern Italy is a dwelling area for more than 17 million humans, with consequent agricultural activities and livestock farming. This area has a Mediterranean continental climate, with an annual average precipitation of 1036 mm and a mean temperature of 12 °C. The largest river basin in Italy, the Po River basin (71,000 km^2^), is included in this area and we focused our investigation on the Po River itself (in all its course), the Oglio River (one of the most important hydrographic left tributaries of the Po River) and the hydrographic right tributaries in the Emilia-Romagna region. We also analyzed two additional groups of rivers outside of this basin: the Brenta River (north-east of the Po River basin) and the Romagna rivers basin (south of the Po River basin). Overall, a total of 335 sampling sites in 105 watercourses were included in this study, covering a wide range of freshwater habitats, different altitudinal zones and environmental conditions (see Supplementary Fig. 1).

Organic material originating from villages, small towns and livestock farms is the main source of river pollution for rivers in the uplands. Conversely, urbanization and intensive agriculture, causing high nutrient loads and consequent eutrophication, are the main factors affecting lowland rivers^10^. A network of drainage canals was established in the lowlands to support agricultural irrigation around the 19^th^ century, with hydrological management aimed at supplying both irrigation and drainage needs^10,47^. The study area was in a late invasion stage^11^ at the time of sampling, since loss of native species and exotic invasion occurred already prior to 1997^10^, before the data analyzed here were collected.

### Data collection

Fish data were collected within monitoring programs of the Emilia-Romagna region^48^, the Padova Province^49^, the Po River^50^ and the Oglio River^51^ over a relatively long-term period (1999–2010). Despite this, community turnover was not a relevant factor in our study, because fish communities are typically stable over such timescales and, despite the ample interval, most of the data were collected over a limited timeframe^44,52^. Fish sampling was performed between spring and autumn by electrofishing, combined with nets in sites of higher water depth and conductivity (e.g. lower stretches of the rivers), further details on fish sampling procedures are described in^8,11^.

Fish species were classified according to^53^, taking into account recent taxonomic determinations and common names as listed in FishBase^54^. Each species was categorized as native or exotic: a species was considered as native when naturally present in a specific basin and as exotic when human-introduced, irrespective of the time elapsed since the introduction. Hybrid specimens or uncertain species were excluded from this study in order to avoid taxonomic asymmetries.

Abundance of each species was expressed with Moyle classes^55^ ranging from 1 (lower abundance, 1–2 individuals per site) to 5 (higher abundance, more than 50 individuals per site). Unfortunately, classes of numerical abundance tend to overestimate the ecological significance of small-bodied species and underestimate that of large-bodied ones, but direct measures of body-mass were not taken at the time of sampling. Attempting to overcome this, a weight was assigned to each species based on their average adult size in the area (1 = small body up to ∼150 g; 2 = medium body ∼150–400 g; 3 = large body over ∼400 g, derived from FishBase and unpublished data) and multiplied by Moyle abundance classes, in order to obtain a body-mass-corrected abundance, hereafter defined simply as abundance^24^.

Water physicochemical sampling was performed with standard methods, in temporal and spatial proximity to the fish sampling, by different Regional Environmental Protection Agencies (*ARPAs*, in Italian) for the Po, the Brenta and the rivers in the Emilia-Romagna region. The Oglio River Water Authority carried out the water sampling in the Oglio River. The geographical position and the elevation of each site were recorded. Eight physicochemical variables were monitored: water temperature, electrical conductivity, chemical oxygen demand, biological oxygen demand, total suspended solids, total phosphorus, ammonia (*NH*_4_^+^) and nitrate nitrogen (*NO*_3_^*−*^).

Land cover data were obtained from the CORINE database (2012, https://www.eea.europa.eu/data-and-maps/data/copernicus-land-monitoring-service-corine). In the lowlands, where estimation of watershed areas is more difficult due to low slopes and human-regulated flow, the land cover of the whole river basin or of the administrative province was used. CORINE land cover classes were merged in five categories based on the main land use in order to better describe the study area: urban use, agricultural use, forest, other natural area, freshwater and brackish water. Land cover was expressed as the share of each of these categories in the watershed of each site.

### Fish functional traits

In order to investigate the functional composition of fish communities, five different ecological functions were examined: feeding, reproduction, migration, tolerance and habitat use. Within these ecological functions, all fish species were classified in guilds, each representing an ecofunctional trait (Supplementary Table 1, see also Noble, *et al*.^56^). Ecological functions, guilds and classification for most species in this study were taken from^24^, where all available information was used to identify appropriate guilds for each species. The same methodology was applied to classify euryhaline species that were not included in previous work (see Supplementary Table 2). A total of 59 fish species were sampled in the study area; of these 37 were native and 22 were exotic species, relatively to the national territory.

### Influence of environmental variables on functional diversity

Fish functional diversity was investigated through the functional dispersion metric FDis,^57^. FDis was calculated using one matrix with species abundance and another matrix of functional traits of fish communities through the *dbFD* function^58^. FDis calculates the spread of traits in multidimensional space by measuring the distance of each species from a centroid weighted by the species abundance.

As a result, FDis measures the relative diversity of functional traits in a community, communities with larger FDis values are more diverse in species traits combinations, whereas low FDis values refer to communities with more traits in common. The advantages in choosing the FDis metric include that it should theoretically not be overly affected by species richness, can be computed from any distance or dissimilarity measures and take into account any number and type of traits (also qualitative traits, as in this study), is not strongly influenced by outliers and accounts for species abundances^57^. FDis was calculated for both the whole fish community and for exotic and native species separately, using species abundance as weights to define the relative representation of each trait in the community.

The relative influence of geographical and land use features, as well as water physicochemical variables, on functional diversity was investigated with a machine learning method: the boosted regression trees (BRT)^59^. BRT analysis is an efficient method to describe any non-linear relationships between variables (e.g. thresholds) and incorporate interactions between variables. Compared to traditional regression methods, BRT analysis combines a large number of simple tree models using the boosting technique to improve the predictive performance. BRT analysis was applied to native and exotic species separately, to investigate differences between the factors affecting the two categories. The relative influence (positive or negative) of each variable was determined by the prevalent direction of its effects on the functional dispersion of species, as no clear unimodal trends were found in our data.

Guild and species abundance matrices for each sampling site were Hellinger transformed^60,61^ to standardize variations among both species and community size, respectively. Environmental variables expressed as percentages were arcsine transformed, while others were log-transformed. All statistical analyses were performed in R software version 3.4.3^62^. FDis was calculated through the homonymous R package^58^. BRT was performed with the ‘gbm’ R package^63^, using standard values (Gaussian distribution, bag fraction of 0.75 and shrinkage of 0.001).

### Impacts of exotic invasions on functional diversity

The degree of exotic invasion in each site was estimated through the abundance of exotic versus native species in each community (% of exotic abundance). This measure was then spatially analyzed for all watercourses within each basin in the study area, through linear kriging^64^.

We also evaluated the spatial overlap and correlation between invasion degree and functional diversity (as expressed through the FDis metric calculated using ecofunctional traits,^65^). First, we used linear kriging to represent functional diversity, based on the whole community, for invaded sites (i.e. where both native and exotic species were present, 198 out of 335 sites, excluding an invaded site at an altitude >400 m a.s.l.). We selected these sites because we could clearly evaluate invasion effects only where both exotic and native species are present, but also, more importantly, because it allowed us to focus our analysis on a geographically uniform area (the lowlands, below 400 m a.s.l.) and thus avoid potentially-confounding geographical factors (i.e. altitude, as species-poor communities at high altitudes typically have low functional diversity, even if not invaded). This area also had a relatively homogeneous fish community at baseline conditions, so that we could consider the functional diversity of least invaded communities as a close proxy to the baseline conditions of reference communities. Following this, we used the same sites to also explore the variations in overall functional diversity along the invasion degree gradient (between 2.3% and 96.5%).

To investigate the impact of exotic invasions on functional diversity we also examined the variations in native and exotic species richness for each stream order, alongside the respective functional diversity of each site, trying to detect patterns indicative of invasion effects. Stream order for each site was calculated using a Digital Elevation Model (DEM) (http://www.sinanet.isprambiente.it/it/sia-ispra/download-mais/dem20/view). Flow direction and accumulation, as well as the watershed of each sampling site, were calculated based on the DEM layer. For the entire river network generated by flow accumulation, stream order was derived with the Strahler method^66^. This procedure was reliable for upland streams, but it was less so in the lowland, possibly due to the fact that flow direction and magnitude in the lowlands are not always natural because of human intervention. The stream order was thus manually checked and revised when necessary in lowland rivers and streams. Rivers were grouped into four classes based on stream order: class 1 (Strahler stream order 1 and 2), class 2 (stream order 3 and 4), class 3 (stream order 5 and 6) and class 4 (stream order > 6). As drainage and irrigation canals could not be assigned into any natural class, a separate class called “Canals” was created. Canals are man-made environments, usually characterized by low habitat heterogeneity and controlled hydrology, located in the lowlands southwest of the Po River, near its delta.

We further examined the relationship between native and exotic richness and their relative functional diversity. We used non-linear regressions, evaluated through a combination of Akaike’s Information Criterion (AIC) weights^67^ and R^2^ values, to estimate best-fitting curves for functional diversity distributions versus species richness and invasion degree. Non-linear best-fitting regressions were estimated using the Curve Expert Professional 2.6 software^68^ and the results of the five best regression models for each relationship were reported in Supplementary Table 3. The spatial analyses were performed with ArcGIS software^69^, using its Kriging Tool and Hydrology Spatial Analyst Tool.

## Supplementary information

Supplementary Info

## Data Availability

Underlying data for this paper is made available also for review as Supplementary Material and will be made publicly available through OpenScienceFramework should the manuscript be accepted for publication.
