# Supplementary material for: Exotic species invasions undermine regional functional diversity of freshwater fish
Source: Sci Rep. 2019 Nov 29;9:17921. doi: 10.1038/s41598-019-54210-1 (PMC6884620; doi:10.1038/s41598-019-54210-1)
Supplement: Supplementary file 1 — Supplementary Info [file 41598_2019_54210_MOESM1_ESM.pdf]

Exotic species invasions undermine regional functional diversity of freshwater fish

Marco Milardi[1], Anna Gavioli[1,*], Janne Soininen[2], Giuseppe Castaldelli[1]

1 - University of Ferrara, Department of Life Sciences and Biotechnology, via Luigi Borsari 46, 44121, Ferrara, Italy. mlrmrc@unife.it, gvlnna@unife.it, ctg@unife.it

2 - University of Helsinki, Department of Geosciences and Geography, PO Box 64, FI-00014, Helsinki, Finland. janne.soininen@helsinki.fi

* Corresponding author

Supplementary Figure 1 – Distribution of the 335 sampling sites used in this study (green dots), the watercourses (in light blue) and the three main basins considered in Northern Italy.

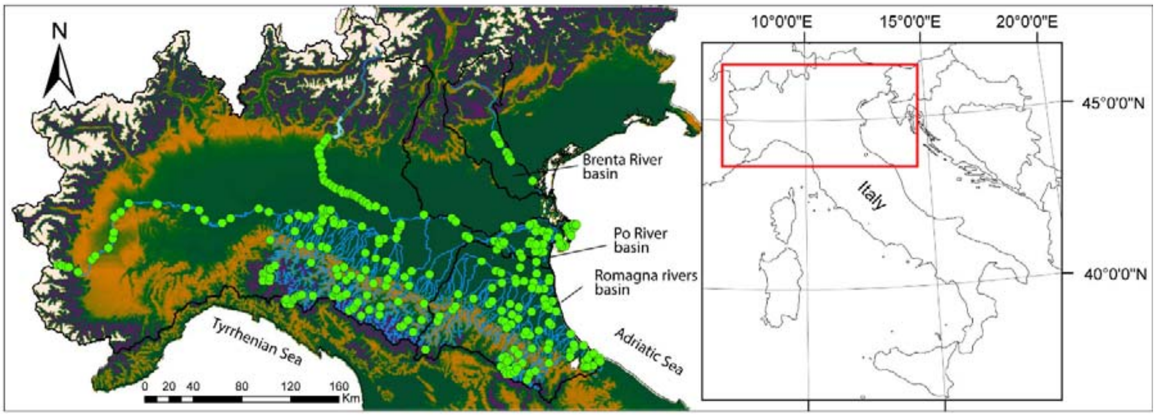

Supplementary Table 1 – Ecological functions and guilds (and their abbreviations) considered for each species in this study. Edited from Milardi and Castaldelli (24).

Ecological function	Guild	Abbreviation	Description
**Feeding**	Planktivores	P	Plankton feeders
	Herbivores	H	Vegetation feeders
	Benthivores	B	Bottom feeders
	Invertivores	I	Invertebrate feeders
	Piscivores	Pi	Fish feeders
	Parasite	Pa	Ematophages
	Generalists	G	Unspecialized feeding
**Reproduction**	Lithophils	Lp	Spawning on stones or gravel
	Phytophils	Pp	Spawning on submerged vegetation
	Phytolithophils	PL	Spawning on stones or vegetation
	Psammophils	PS	Spawning on sand or mud
	Ostracophils	O	Spawning in molluscs
	Pelagophils or live breeding	LB	Pelagic spawners or live spawners
	Polyphils	Pps	Generalist spawners
	Sea spawning	Sea	Saltwater spawners
**Migration**	Short	S	Within or close to the site
	Intermediate	M	Up and downstream or into flooded areas
	Long	L	Anadromous and catadromous species
**Tolerance**	Low oxygen tolerants	OxT	Tolerance/intolerance to low oxygen
	Low oxygen intolerants	OxInt	(indicatively below 3 ppm)
	High temperature tolerants	HTT	Tolerance/intolerance to high temperature
	High temperature intolerants	HTInt	(indicatively above 20 °C)
**Habitat use**	Rheophiles	Rhe	Preferring fast flowing water
	Limnophiles	Lim	Preferring slow or no current
	Eurytopics	Eur	Having no preference on current velocity
	Clear water	CW	Clear water adapted
	Turbid water	TW	Turbid water adapted
	Wide range of conditions	WR	Adapted to a wide range of water turbidity

Supplementary Table 2: Ecological functions and guilds of each species sampled in this study.

					Ecological function: Feeding							Reproduction								Migration			Tolerance				Habitat use						Status		Body size		
					Planktivores	Herbivores	Benthivores	Invertivores	Piscivores	Parasite	Generalists	Lithophils	Phytophils	Phytolithophils	Psammophils	Ostracophils	Pelagophils or live breeding	Polyphils	Sea spawning	Short	Intermediate	Long (anadromous or catadromous)	Low oxygen tolerant	Low oxygen intolerant	High temperature tolerant	High temperature intolerant	Rheophiles	Limnophiles	Eurytopic	Clear water	Turbid water	Wide range of conditions	Native	Exotic	Small	Medium	Large
	**Abbr.**	**Common name**	**Family**	**Species**	P	H	B	I	Pi	Pa	G	Lp	Pp	PL	PS	O	LB	Pps	Sea	S	M	L	OxT	OxInt	HT	HinT	Rhe	Lim	Eur	CW	TW	WR			1	2	3
1	Aang	European eel	Anguillidae	*Anguilla anguilla*					Pi										Sea			L	OxT		HT		Rhe					WR	N			2	
2	Afal	Twaite shad	Clupeidae	*Alosa fallax*					Pi			Lp										L		OxInt		HinT	Rhe					WR	N			2	
3	Srub	South European roach	Cyprinidae	*Sarmarutilus rubilio*							G			PL						S			OxT		HT				Eur	CW			N		1		
4	Laul	Italian red-eye roach	Cyprinidae	*Leucos aula*							G		Pp							S			OxT		HT			Lim				WR	N		1		
5	Ssqu	Italian chub	Cyprinidae	*Squalius squalus*				I				Lp									M		OxT		HT				Eur			WR	N			2	
6	Ttin	Tench	Cyprinidae	*Tinca tinca*				I					Pp								M		OxT		HT			Lim		CW			N			2	
7	Shes	Italian rudd	Cyprinidae	*Scardinius hesperidicus*							G			PL						S			OxT		HT				Eur			WR	N		1		
8	Aarb	Italian bleak	Cyprinidae	*Alburnus arborella*				I						PL						S				OxInt		HinT			Eur			WR	N		1		
9	Csoe	Italian nase	Cyprinidae	*Chondrostoma soetta*				I				Lp									M			OxInt		HinT		Lim		CW			N			2	
10	Pgen	South-european nase	Cyprinidae	*Protochondrostoma genei*				I				Lp									M			OxInt		HinT	Rhe			CW			N			2	
11	Bple	Italian barbel	Cyprinidae	*Barbus plebejus*			B					Lp								S				OxInt		HinT	Rhe			CW			N		1		
12	Cbil	Italian spined-loach	Cobitidae	*Cobitis bilineata*			B					Lp								S				OxInt		HinT	Rhe			CW			N		1		
13	Ecis	Southern pike	Esocidae	*Esox cisalpinus*					Pi				Pp							S			OxT			HinT		Lim		CW			N				3
14	Cgob	Bullhead	Cottidae	*Cottus gobio*			B					Lp								S				OxInt		HinT	Rhe			CW			N		1		
15	Pflu	European perch	Percidae	*Perca fluviatilis*					Pi				Pp							S			OxT			HinT			Eur	CW			N			2	
16	Lram	Thinlip mullet	Mugilidae	*Liza ramada*							G								Sea		M		OxT		HT				Eur			WR	N			2	
17	Pbon	Padanian goby	Gobiidae	*Padogobius bonelli*			B					Lp								S				OxInt		HinT	Rhe			CW			N		1		
18	Mcep	Flathead grey mullet	Mugilidae	*Mugil cephalus*							G								Sea		M		OxT		HT				Eur			WR	N			2	
19	Eenc	European anchovy	Engraulidae	Engraulis encrasicolus	P														Sea		M		OxT		HT				Eur			WR	N		1		
20	Laur	Golden grey mullet	Mugilidae	*Liza aurata*							**G**								Sea		M		OxT		HT				Eur			WR	N			2	
21	Lsal	Leaping mullet	Mugilidae	*Liza saliens*							G								Sea		M		OxT		HT				Eur			WR	N			2	
22	Sflu	Freshwater blenny	Blennidae	*Salaria fluviatilis*			B					Lp							Sea	S				OxInt		HinT	Rhe			CW			N		1		
23	Aboy	Big-scale sand smelt	Atherinidae	*Atherina boyeri*							G						LB				M		OxT		HT							WR	N		1		
24	Lami	Leerfish	Carangidae	*Lichia amia*					**Pi**										Sea		M			OxInt		HinT			Eur			WR	N				3
25	Afas	Mediterranean banded killi	Cyprinodontidae	*Aphanius fasciatus*				I										Pps		S			OxT		HT			Lim		CW			N		1		
26	Ucir	Shi drum	Sciaenidae	*Umbrina cirrosa*			B												Sea		M		OxT		HT				Eur			WR	N				3
27	Rpig	Pigo	Cyprinidae	*Rutilus pigus*							G			PL						S				OxInt		HinT			Eur			WR	N			2	
28	Pfle	European flounder	Pleuronectidae	*Platichthys flesus*			B												Sea		M		OxT		HT				Eur			WR	N		1		
29	Bcan	Brook barbel	Cyprinidae	*Barbus caninus*			B					Lp								S				OxInt		HinT	Rhe					WR	N		1		
30	Tmut	Italian riffle dace	Cyprinidae	*Telestes muticellus*							G			PL						S				OxInt		HinT			Eur		TW		N		1		
31	Ppho	Eurasian minnow	Cyprinidae	*Phoxinus phoxinus*							G	Lp									M		OxT		HT				Eur	CW			N		1		
32	Rben	Italian gudgeon	Gobiidae	*Romanogobio benacensis*			B					Lp								S				OxInt		HinT	Rhe			CW			N		1		
33	Stco	Brown trout	Salmonidae	*Salmo trutta*				I				Lp									M			OxInt		HinT	Rhe			CW			N			2	
34	Smar	Marble trout	Salmonidae	*Salmo marmoratus*				I				Lp									M			OxInt		HinT	Rhe			CW			N				3
35	Gacu	Three-spined stickleback	Gasterosteidae	*Gasterosteus aculeatus*				I					Pp							S			OxT		HT			Lim				WR		E	1		
36	Tthy	Grayling	Salmonidae	*Thymallus thymallus*				I				Lp									M			OxInt		HinT	Rhe			CW			N		1		
37	Lzan	Po brook lamprey	Petromyzontidae	*Lethenteron zanandreai*						Pa		Lp									M			OxInt		HinT	Rhe					WR	N			2	
38	Kpun	Italian spring goby	Gobiidae	*Knipowitschia punctatissima*			B					Lp								S				OxInt		HinT	Rhe				TW		N		1		
39	Bbar	Barbel	Cyprinidae	*Barbus barbus*			B					Lp								S				OxInt	HT		Rhe					WR	N			2	
40	Cspc	Crucian carp	Cyprinidae	*Carassius carassius*							G			PL						S			OxT		HT				Eur		TW			E	1		
41	Ccar	Common carp	Cyprinidae	*Cyprinus carpio*			B						Pp							S			OxT		HT				Eur		TW			E			3
42	Bbjo	White bream	Cyprinidae	*Blicca bjoerkna*							G		Pp							S			OxT		HT				Eur		TW			E		2	
43	Abra	Freshwater bream	Cyprinidae	*Abramis brama*			B						Pp							S			OxT		HT				Eur		TW			E		2	
44	Rrut	Roach	Cyprinidae	*Rutilus rutilus*							G			PL						S			OxT		HT				Eur			WR		E	1		
45	Rser	Bitterling	Cyprinidae	*Rhodeus sericeus*							G					O				S			OxT		HT			Lim				WR		E	1		
46	Ppar	Stone moroko	Cyprinidae	*Pseudorasbora parva*				I				Lp								S			OxT		HT				Eur			WR		E	1		
47	Cide	Grass carp	Cyprinidae	*Ctenopharyngodon idella*		H											LB				M		OxT			HinT			Eur		TW			E			3
48	Lasp	Asp	Cyprinidae	*Leuciscus aspius*					Pi					PL							M		OxT			HinT	Rhe				TW			E		2	
49	Sgla	Wels catfish	Siluridae	*Silurus glanis*					Pi						PS					S			OxT		HT				Eur		TW			E			3
50	Amel	Black bullhead	Ictaluridae	*Ameiurus melas*			B								PS					S			OxT		HT			Lim			TW			E		2	
51	Ipun	Channel catfish	Ictaluridae	*Ictalurus punctatus*					Pi						PS					S			OxT		HT				Eur		TW			E		2	
52	Ghol	Eastern mosquitofish	Cyprinodontidae	*Gambusia holbrooki*				I										Pps		S			OxT		HT			Lim				WR		E	1		
53	Msal	Largemouth black bass	Centrarchidae	*Micropterus salmoides*					Pi						PS					S			OxT		HT			Lim		CW				E		2	
54	Lgib	Pumpkinseed	Centrarchidae	*Lepomis gibbosus*				I										Pps		S			OxT		HT			Lim		CW				E	1		
55	Zluc	Pike-perch	Percidae	*Sander lucioperca*					Pi						PS					S			OxT		HT			Lim			TW			E		2	
56	Gcer	Ruffe	Percidae	*Gymnocephalus cernua*				I						PL						S			OxT		HT			Lim				WR		E	1		
57	Mang	Pond loach	Cobitidae	*Misgurnus anguillicaudatus*			B					Lp								S			OxT		HT		Rhe					WR		E	1		
58	Omyk	Rainbow trout	Salmonidae	*Oncorhynchus mykiss*				I				Lp									M			OxInt		HinT	Rhe			CW				E		2	
59	Scep	Chub	Cyprinidae	*Squalius cephalus*				I				Lp									M		OxT		HT				Eur			WR		E		2	

Supplementary Table 3 – Evaluation of best fit for regression equations through a combination of AIC weights (W AIC) and R^2 (or pseudo R^2) values. Only the best 5 fitting regressions are included for each relationship, in decreasing order of best fit. (a) Functional diversity vs invasion degree (Figure 3), (b) Functional diversity vs exotic species richness and (c) Functional diversity vs native species richness (Figure 5).

(a)

Regression	Equation	N of parameters	R^2 (pseudo R^2)	AIC	ΔAIC	W AIC
Ratkowski Model	y = a/(1+exp(b-c*x))	3	.448	-1187.52	0	.409
Rational Model	y = (a+b*x)/(1+c*x+d*x^2)	4	.448	-1185.48	2.04	.147
DR-Hill	y = alpha + theta*x^eta/(kappa^eta + x^eta)	3	.446	-1186.77	0.75	.281
Logistic Power	y = a/(1+(x/b)^c)	3	.446	-1186.77	0.75	.281
Bleasdale-YD	y = x*(a+b*x^(-theta))^(-1/theta)	3	.446	-1186.83	0.69	.289

(b)

Regression	Equation	N of parameters	R^2 (pseudo R^2)	AIC	ΔAIC	W AIC
Heat Capacity	y = a + b*x + c/x^2	3	.8	-1249.64	0	3.393038707
Exponential Plus Linear	y = a+br^x+c*x	4	.799	-1246.77	2.87	.807934647
MMF	y = (ab+cx^d)/(b+x^d)	4	.794	-1242.14	7.5	.079796622
Rational Model	y = (a+b*x)/(1+c*x+d*x^2)	4	.794	-1241.64	8	.062145672
Weibull	y = a-be^-cx^d	4	.793	-1241.21	8.43	.05012306

(c)

Regression	Equation	N of parameters	R^2 (pseudo R^2)	AIC	ΔAIC	W AIC
Root	y = ab^1/x	2	.448	-1403.73	0	.409
Modified Hoerl	y = (ab^1/x)*(x^c)	3	.448	-1401.87	1.86	.161
MMF	y = (ab+cx^d)/(b+x^d)	4	.448	-1399.98	3.75	.062
Rational Model	y = (a+b*x)/(1+c*x+d*x^2)	4	.449	-1399.95	3.78	.178
Weibull	y = a-be^-cx^d	4	.448	-1399.9	3.83	.060
